# Effect of Combined Training on Body Image, Body Composition and Functional Capacity in Patients with Breast Cancer: Controlled Clinical Trial

**DOI:** 10.1055/s-0043-1770126

**Published:** 2023-06-20

**Authors:** Andréa Dias Reis, Paula Tâmara Vieira Teixeira Pereira, Jurema Gonçalves Lopes Castro Filha, Evelyn Feitosa Rodrigues, Isadora Pinheiro Laranjeira, Bianca Trovello Ramallo, Marcela Rodrigues de Castro, Fabrício Eduardo Rossi, Ismael Forte Freitas Júnior, João Batista Santos Garcia

**Affiliations:** 1 Universidade do Oeste Paulista, Presidente Prudente, SP, Brazil; 2 Universidade Federal do Maranhão, São Luís, MA, Brazil; 3 Universidade São Judas Tadeu, São Paulo, SP, Brazil; 4 Universidade Federal da Bahia, Salvador, BA, Brazil; 5 Universidade Federal do Piauí, Teresina, PI, Brazil

**Keywords:** Breast neoplasms, Health, Exercise, Women, Neoplasias da mama, Saúde, Exercício, Mulheres

## Abstract

**Objective:** Evaluate the effect of combined training on body image (BI), body composition and functional capacity in patients with breast cancer. As also the relationship of BI with body composition and functional capacity.

**Methods:** This was a Controlled Clinical Trial study, this study including 26 patients with breast cancer (30 to 59 years). The training group (n = 13) underwent 12 weeks of training, including three 60-min sessions of aerobic exercise and resistance training, and two sessions of flexibility training per week; each flexibility exercise lasted 20s. The Control Group (n = 13) received only the standard hospital treatment. Participants were evaluated at baseline and after 12 weeks. BI (primary outcomes) was assessed using the Body Image After Breast Cancer Questionnaire; Body composition was estimated with the indicators: Body mass index; Weight, Waist hip Ratio; Waist height ratio; Conicity index; Reciprocal ponderal index; Percentage of fat; Circumference of the abdomen and waist; Functional capacity by cardiorespiratory fitness (cycle ergometer) and strength (manual dynamometer). The statistic was performed in the Biostatistics and Stata 14.0 (α = 5%).

**Results:** The patients in the training group showed a reduction in the limitation dimension (p = 0.036) on BI, However, an increase in waist circumference was observed in both groups. In addition an increase in VO2max (p < 0.001) and strength in the right (p = 0.005) and left arms (p = 0.033).

**Conclusion:** Combined training demonstrates to be an effective and non-pharmacological strategy to patients with breast cancer, with improvement on BI and functional capacity, changing related variables negatively when there is no physical training.

## Resumo

**Objetivo:** Avaliar o efeito do treinamento combinado na imagem corporal (IB), composição corporal e capacidade funcional em pacientes com câncer de mama. Assim como a relação do IB com a composição corporal e capacidade funcional.

**Métodos:** Este foi um estudo de Ensaio Clínico Controlado, este estudo incluiu 26 pacientes com câncer de mama (30 a 59 anos). O grupo de treinamento (n = 13) foi submetido a 12 semanas de treinamento, incluindo três sessões de 60 min de exercício aeróbio e treinamento de resistência, e duas sessões de treinamento de flexibilidade por semana; cada exercício de flexibilidade durou 20s. O Grupo Controle (n = 13) recebeu apenas o tratamento hospitalar padrão. Os participantes foram avaliados no início e após 12 semanas. O IB (desfechos primários) foi avaliado por meio do Body Image After Breast Cancer Questionnaire; A composição corporal foi estimada com os indicadores: índice de massa corporal; Peso, relação cintura-quadril; Relação da altura da cintura; Índice de conicidade; Índice ponderal recíproco; Porcentagem de gordura; Circunferência do abdômen e cintura; Capacidade funcional por aptidão cardiorrespiratória (cicloergômetro) e força (dinamômetro manual). A estatística foi realizada na Bioestatística e no Stata 14.0 (α = 5%).

**Resultados:** Os pacientes do grupo de treinamento apresentaram redução da dimensão da limitação (p = 0,036) no IB, porém, foi observado aumento da circunferência da cintura em ambos os grupos. Além disso, um aumento do VO2máx (p <0,001) e da força nos braços direito (p = 0,005) e esquerdo (p = 0,033).

**Conclusão:** O treinamento combinado demonstra ser uma estratégia eficaz e não farmacológica para pacientes com câncer de mama, com melhora do IB e da capacidade funcional, alterando variáveis relacionadas negativamente quando não há treinamento físico.

## Introduction

At least 16% of the world's population died of cancer in 2015, with breast cancer being the most frequent in women, especially in underdeveloped countries. 1 The disease and its treatment can generate important changes in body appearance and functionality. Changes in appearance include alopecia, surgical scars, breast removal, rashes etc. 2 3 Mastectomy can lead to emotional, social, postural, and sexual alterations. It can also cause lesions in muscles, lymphedema, and a decrease or loss in the range of motion. Furthermore, patients on antineoplastic therapy may present reduced strength and cardiopulmonary capacity. 4

Body composition is considered a worrying factor, since treatment may imply a weight gain of 71.43%. 5 Excess weight and obesity are a poor prognosis, due to increases in tumors, the positivity of estrogen and progesterone receptors, risk of distant metastasis, and mortality. 6

There has been an expansion in the clinical and investigative interest in oncology since 2012, 7 when Supportive Care in Cancer highlighted the importance of assessing body image (BI) in cancer patients. BI is a multidimensional construct, and it is necessary to consider the subjective experiences of the disease, 3 as well as the symbolic value attributed to specific body segments, such as the breast, for the woman. 7

Thus, in the elaboration of BI, the social and cultural influence derived from interpersonal experiences should be considered, allied to several elements of the appearance and body functionality. 8 Therefore, women with breast cancer are vulnerable to adverse impacts on their BI 9 10 which in turn can generate various consequences such as anxiety, depression, mutilation, and low self-esteem. 11

Physical training methods, both strength and aerobic, have been used as safe and well tolerated interventions in cancer patients. 12 13 14 Studies have shown a decrease in body fat, and improvements in cardiopulmonary function, strength, 12 13 and BI 15 16 after interventions with physical activity. However, although interventions with combined training (CT), aerobic and resistance training in the same session, are widely used, 17 18 19 the available information is still insipid. 13 Little is known about the influence of combined training (underwent 12 weeks of training, including three 60-min sessions of aerobic exercise and resistance training, and two sessions of flexibility training per week; each flexibility exercise lasted 20 s and was performed in sets of three repetitions) on BI, on functional and body composition parameters in this population.

In addition, the investigation of parameters in the construction of BI, appearance and function, with elements of body composition and functional capacity in patients with breast cancer, since suffer adverse effects in cancer treatment. Thus, the present study aims to evaluate the influence of CT on the BI, body composition and functional capacity of patients with breast cancer, as also the relationship of BI with body composition and functional capacity.

## Methods

### Study Design

Controlled Clinical Trial study, intervention training combined (aerobic, resistance and flexibility training) the 12 weeks in patients with breast cancer.

### Participants

Thirty-one women (30 to 59 years old) selected in the Hospital participated in the study, through standardized invitations given at routine meetings at the institution. The inclusion criteria were: 1)Not having performed physical training for at least 6 months, 2)Being on treatment (chemotherapy and hormone therapy, radiotherapy) or monitoring of breast neoplasms, 3)No diagnosis of mental disorders or psychological disorders, 4)Able to communicate verbally, 5)No motor restrictions, 6)Not pregnant or lactating, 7)Performing all evaluations, 8)Having previous medical release. To remain in the physical training group, it was necessary to not be absent from more than three consecutive sessions.

Patients were contacted and invited to participate in this study by telephone, through invitations issued at regularly-scheduled meetings with HCAB patients, and by referral from oncologists, mastologists, physiatrists, physical therapists, psychologists and pain management specialists. Patients who showed interest received a complete explanation of the study.

Groups were divided 1:1, they were randomly with sweepstakes, assigned to groups into the Training Group (TG), with 15 patients who performed CT for 12 weeks together with conventional hospital treatment, and the Control Group (CG) with 16 patients who only underwent conventional hospital treatment (chemotherapy and hormone therapy, radiotherapy) for 12 weeks. The physical evaluations were performed blindly by the evaluator, who was only informed of the day and time of evaluations. The sample size was calculated using statistical G-power 3.1, with power 0.8 and level of significance 0.05, which showed that twelve participants were needed.

Participants were informed about the objectives of the study and written informed consent was obtained. The study had approval from the Research Ethics Committee of the Federal University of Maranhão, protocol number 20665713.2.0000.5087 and Trial Registration: NCT03061773.

Assessments of both the TG and the CG were conducted at the study's outset to establish a baseline, and at the end of 12 weeks, corresponding to the length of the combined training intervention. The team was trained in the application of each survey and test procedure, and the researchers were blinded with regards to the physical assessments, only being informed of the day and time of the assessments.

### Intervention

The CT program consisted of aerobic, resistance, and flexibility exercises lasting 12 weeks, with 3 sessions per week of aerobic and resistance training in the same session (supervised by trainers specialized in physical exercise) and intercalate 2 sessions per week of flexibility training. Each aerobic and resistance training session lasted 60 minutes, following the order: 30 minutes on cycle ergometer, hip flexion and extension, shoulder development, Swiss ball squatting, French triceps, and curved paddling.

The aerobic training was controlled by the training heart rate. 20 21 22 23 24 25 26 In the cardiorespiratory test, the ramp protocol adapted. 27 was used on a cycle ergometer (ERGO FIT brand, model ERGO 167-FITC CYCLE). Blood pressure was measured with conventional mercury column apparatus (BD®), heart rate (Polar FT2) and subjective perception of exertion using the Borg scale (Inforfisic Mark) in the final 15 seconds of the stages. Before and after the cardiorespiratory test, the patients remained seated at rest to verify the subjective perception of exertion, blood pressure, and heart rate. The test was performed after a 72-hour interval of familiarization. subjective perception of exertion was used to verify the individualized intensity of training (7 to 20), with the patients verbally encouraged to reach maximum fatigue.

The load progressions were performed every 4 weeks, respecting the biological individuality in the cardiorespiratory capacity test and maximal repetitions to predict the initial load. 28 The initial intensity of the aerobic training was 50 to 60% of the training heart rate, ending with 80 to 90% of the training heart rate. The load of the resistance training started with the weight of the body itself or 1kg in dumbbells and shin guards, and moderate intensity in the elastic band. In the fifth week, there was an increase to 1kg and strong intensity in the elastic band, remaining until the twelfth week.

The resistance training protocol included 3 sets for each exercise with 12 repetitions and a one-minute interval between sets and repetitions. The speed of execution of each movement was three seconds in the concentric phase and three seconds in the eccentric phase. 29 The exercises were alternated by segment, prioritizing the large muscle groups. The loads were by means of shin guards, dumbbells, elastic bands, and the weight of the body itself.

The resistance training load was verified by means of the maximal repetition test, with 12 repetitions and a 72-hour interval of familiarization. 30 Patients who exceeded 12 repetitions were given a 5-minute interval before performing the 12 repetitions with a new load. The flexibility training was active, without pain, where each exercise lasted 20 seconds in 3 series. 28 Participants were instructed to perform ten stretches.

## Primary Outcomes Measures

### Body Image

BI was assessed using the Body Image After Breast Cancer Questionnaire, 20 a specific instrument for patients with breast cancer. This tool was validated for the Brazilian female audience. 3 It consists of 44 questions organized in 6 dimensions: 1)Vulnerability (V), 2)Transparency (T), 3)Body Stigma (BS), 4)Concerns about the Arm (CA), 5) Body Concerns (BC), and 6)Limitations (L). The answers are given on a *Likert* scale of agreement (1 to 5). The scores vary according to the scale and surgery; the higher the score, the more compromised the BI. 3 21 For the questions that presented negative scores, a value of 6 is inserted for the calculations of the dimensions.

## Secondary Outcomes Measures

### Body Composition

Anthropometric measurements were taken on a mechanical scale (Fillizola®, São Paulo, Brazil) to the nearest 0.1 kg, on a fixed stadiometer (Sanny®, São Paulo, Brazil) to the nearest 0.1 cm and with a measuring tape (Sanny®, São Paulo, Brazil) to the nearest 0.1 cm respectively. 22 The weight was measured with the women without moving with the weight distributed equally between the feet in the center of the platform of the electronic scale, the participants. 22 Height was measured with the participants in the Frankfurt plan and stared at the horizon. 22 The circumference and skinfold measurements were verified with the participants standing and relaxed muscles. 22

Body composition was verified through the following indicators: 2)Weight 22 ; 1)Body Mass Index (BMI) 23 ; 3)Waist Hip Ratio 24 ; 4)Waist Height Ratio 24 ; 5)Conicity Index 24 ; 6)Reciprocal Ponderal Index 25 ; 7)Percentage of Fat 23 ; 8)Circumference of the abdomen, waist (WC), hip, and right and left thighs 22 ; 9)Fat-free mass. 22

### Functional Capacity

The static force was evaluated by grip strength using a hand held dynamometer (Jamar Sammons Preston) scale from 0 to 100 kilograms. Guidance was provided to press the equipment with maximum force, without flexing the elbow or changing the posture. 22 The participants were seated with the adducted shoulder and turned in a neutral way, elbow flexed at 90° and forearm and wrist in a neutral position. 31 Three attempts were allowed on both sides (alternately) and select the best result. 22 The maximum oxygen volume (VO2max) was measured using the estimated submaximal cycle ergometer test 23 based on the final power in a protocol of 15 Watts per minute, using the formula for women.

### Statistical Analysis

Data normality was checked using the Shapiro-Wilk and Kolmogorov-Smirnov tests. The comparisons of the baseline variables between groups were analyzed using the Student t test for independent samples when parametric statistics were observed. If the data presented non-parametric distribution, the Mann-Whitney and dichotomous variables χ ^2^ and Fisher's Exact test were used.

The differences between the CT and control groups were analyzed by two-way repeated measures of ANOVA (group x time). When a signiﬁcant interaction was observed, a Bonferroni post hoc test was conducted. For all measured variables the estimated sphericity was veriﬁed according to Mauchly's W test and the Greenhouse–Geisser correction when necessary. The partial eta-squared was classified according to Cohen. 32 The correlation was verified through the tests: Pearson and Spearman, with classifications. 33 The data were analyzed using the Biostatistics and Stata 14.0, significance was set at p < 0.05.

## Results

**Fig. 1:**
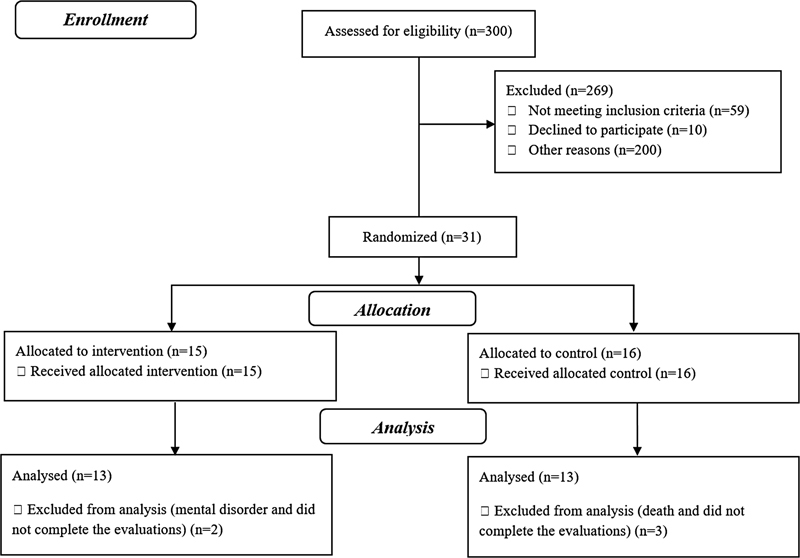
Consort flowchart.

Twenty-six patients (13 TG and 13 CG) completed the study ( Figure 1 ). The patients presented homogeneity between the groups ( Table 1 ). Regarding the clinical aspects, only one patient in the TG had a bilateral mastectomy and patients of neither group performed breast reconstructions.

**Table 1:** Characteristics of patients with breast cancer

Variables	Training group	Control group	p-value
Antopométricas a			
Age (years)	46.9 ± 7.4	51.8 ± 12.5	0.303
Height (m)	1.5 ± 0.1	1.5 ± 0.1	0.962
Weight (kg)	58.2 ± 9.7	63.2 ± 11.4	0.200
Employed ^b^			
Yes	4(30.77%)	0	0.057
No	9(69.23%)	13(100%)	
Marital Status ^b^			
Single	7(53.85%)	6(46.15%)	0.848
Married	6(46.15%)	7(53.85%)	
Time since most recent physical training ^b^			
3 to 12 months	4(30.77)	0	0.057
> 12 months	9(69.23)	13(100)	
Clinical Period ^b^			
Observation	6(46.15%)	2(15.38%)	0.343
Chemotherapy and Hormone Therapy	5(38.46%)	8(61.54%)	
Radiation Therapy	2(15.38%)	3(23.08%)	
Type of Tumor ^b^			
Invasive Ductal Carcinoma	13 (100%)	11(84.62%)	0.220
Fuso-cellular or Epithelial Carcinoma	0	2(15.38%)	
Typo of Surgery ^b^			
Conservative	3(23.08)	2(15.38)	0.500
Mastectomy	10 (76.92)	11(84.62)	
Mastectomy + Reconstruction	0	0	

a Student T-Test; ^b^ Fisher's Exact; ^d^ Chi-squared test; * p < 0.05; Expressed values: mean ± standard deviation. absolute frequency (relative frequency).

BI of the patients who performed CT presented a reduction for the L dimension (p = 0.036). In the concern with the body dimension was a tendency for TG reduction (Δ = 5.23, p = 0.183). In the transparency dimension, the groups presented differences (p = 0.009). In the analysis of body composition, a change was observed after 12 weeks for WC (p = 0.034), fat-free mass (p = 0.032) and circumference of the right thigh (p = 0.049). Strength presented changes after 12 weeks, for both the right arm (p = 0.005) and observed left arm (p = 0.033). The detected increased right arm strength for the TG (p = < 0.001). In the VO2max there was also changes after 12 weeks (p = < 0.001) ( Table 2 ).

**Table 2:** Body image, body composition and functional capacity of patients with breast cancer undergoing combined training

Variables	TG (n = 13)		CG (n = 13)						
	Base	12 weeks	Base	12 weeks	Time	Group	Interaction	Eta-squared
Body image								
Body stigma	32.5 ± 10.6	33.1 ± 7.7	36.1 ± 12.4	35.6 ± 14.3	0.981	0.478	0.759	0.00
Limitations	16.2 ± 4.8	11.2 ± 2.9*	13.9 ± 6.7	14.9 ± 5.7	0.036	0.704	0.003	0.20
Concerns about the arm	8.7 ± 3.9	9.8 ± 4.1	8.2 ± 3.2	9.8 ± 3.8	0.251	0.773	0.816	0.05
Body concerns	17.9 ± 7.0	12.7 ± 3.8	14.3 ± 6.0	15.9 ± 6.8	0.183	0.906	0.019	0.10
Transparency	14.7 ± 5.0	12.1 ± 3.3	17.8 ± 5.2	18.0 ± 5.9	0.295	0.009	0.214	0.04
Vulnerability	24.2 ± 9.1	19.4 ± 4.2	21.9 ± 9.6	22.7 ± 8.4	0.198	0.870	0.071	0.10
Body composition								
Body mass index (Kg/m ^2^ )	24.6 ± 3.7	24.6 ± 2.9	26.8 ± 4.0	26.8 ± 3.9	0.864	0.135	0.947	0.00
Weight (kg)	58.2 ± 9.7	58.9 ± 9.2	63.2 ± 11.4	63.4 ± 10.9	0.224	0.257	0.479	0.10
Waist hip ratio (cm/cm)	0.8 ± 0.0	0.8 ± 0.0	0.8 ± 0.1	0.8 ± 0.1	0.112	0.086	0.702	0.10
Waist height ratio (cm/cm)	0.5 ± 0.0	0.5 ± 0.0	0.5 ± 0.1	0.5 ± 0.1	0.259	0.071	0.689	0.05
Conicity index (m/kg/m)	1.2 ± 0.0	1.2 ± 0.0	1.2 ± 0.2	1.3 ± 0.7	0.121	0.075	0.457	0.10
Reciprocal ponderal index (cm/kg)	39.8 ± 2.0	39.9 ± 1.6	38.7 ± 2.0	38.7 ± 1.8	0.845	0.112	0.860	0.00
Percentage of fat (%)	28.5 ± 5.6	27.4 ± 5.6	28.5 ± 5.6	27.4 ± 5.6	0.124	1.000	1.000	0.10
Circumference of the abdômen (cm)	87.1 ± 7.2	88.2 ± 8.0	92.2 ± 8.8	92.7 ± 9.4	0.191	0.148	0.626	0.10
Circumference of the waist (cm)	77.3 ± 6.5	78.8 ± 6.7	82.7 ± 8.9	83.3 ± 8.4	0.034	0.110	0.336	0.20
Circumference of the hip (cm)	96.9 ± 8.2	97.5 ± 6.7	100.0 ± 9.6	99.9 ± 8.3	0.655	0.408	0.553	0.01
Circumference of the right thigh (cm)	56.2 ± 6.6	53.7 ± 7.1	50.9 ± 3.9	51.9 ± 5.1	0.417	0.102	0.049	0.03
Circumference of the left thigh (cm)	54.5 ± 9.1	52.8 ± 7.2	50.4 ± 4.1	51.8 ± 5.5	0.897	0.298	0.173	0.00
Fat-free mass (Kg)	41.3 ± 4.8	42.4 ± 3.9	45.0 ± 7.6	45.8 ± 7.5	0.032	0.147	0.781	0.20
Functional capacity								
Strength of the right arm (Kgf)	20.2 ± 6.1	25.7 ± 5.5*	21.4 ± 7.4	20.5 ± 7.1	0.005	0.420	<0.001	0.30
Strength of the left arm (Kgf)	20.2 ± 6.2	23.4 ± 5.3	22.8 ± 6.9	22.2 ± 6.8	0.033	0.765	0.004	0.20
Maximum oxygen volume (ml ^−1^ kg ^−1^ min ^−1^ )	16.9 ± 2.0	20.7 ± 2.6 ^#^	11.9 ± 3.0	14.9 ± 3.0	<0.001	<0.001	0.243	0.51

*Difference in the TG after 12 weeks of training; ^#^ Difference between groups; TG: Training Group; CG: Control Group; Time: time referring to the intervention period; Interaction: Referring to the interaction between time and group.

At the baseline period, the CG demonstrated a strong negative correlation between WC and all BI dimensions. After 12 weeks, the fat percentage was still correlated strongly and positively with the BC (p = 0.016). The muscle strength of the right arm was negatively and moderately correlated in the basal period with the L (p = 0.042) and after 12 weeks it remained negative, but strong, for the L (p = 0.002) and moderate for the CA(0.019) and T (p = 0.035) ( Table 3 ).

**Table 3:** Correlation between body image, body composition and functional capacity of patients with breast cancer undergoing combined training

Variables	TG				CG			
	Base		12 weeks		Base		12 weeks	
Corporal Stigma Dimension	r	p-value	r	p-value	r	p-value	r	p-value
Body mass index (Kg/m ^2^ )	0.03	0.925	0.17	0.581	0.54	0.057	0.20	0.519
Weight (kg)	-0.04	0.897	-0.05	0.867	0.48	0.101	0.16	0.612
Waist hip ratio (cm/cm)	0.24	0.431	0.13	0.676	-0.05	0.865	0.15	0.627
Percentage of fat (%)	-0.22	0.469	-0.06	0.857	0.59	0.035 ^b^	0.35	0.235
Circumference of the waist (cm)	0.03	0.915	0.02	0.954	-0.66	0.014 a	0.20	0.509
Fat-free mass (Kg)	0.14	0.649	0.01	1.000	0.21	0.489	-0.02	0.954
Strength of the right arm (Kgf)	-0.31	0.309	-0.09	0.780	0.31	0.307	-0.17	0.580
Strength of the left arm (Kgf)	-0.34	0.261	-0.14	0.655	0.40	0.181	-0.06	0.851
Maximum oxygen volume (ml ^−1^ kg ^−1^ min ^−1^ )	0.20	0.509	0.14	0.648	-0.49	0.091	0.09	0.782
**Limitations Dimension**								
Body mass index (Kg/m ^2^ )	0.16	0.610	-0.442	0.131	0.01	0.998	0.01	0.991
Weight (kg)	0.24	0.431	-0.291	0.335	0.03	0.928	0.19	0.536
Waist hip ratio (cm/cm)	-0.02	0.938	-0.459	0.115	-0.15	0.616	0.04	0.891
Percentage of fat (%)	-0.09	0.768	-0.268	0.377	0.52	0.066	0.33	0.270
Circumference of the waist (cm)	0.16	0.608	-0.385	0.194	-0.66	0.014 a	-0.37	0.210
Fat-free mass (Kg)	0.33	0.266	-0.239	0.431	-0.22	0.477	0.03	0.926
Strength of the right arm (Kgf)	-0.41	0.167	-0.233	0.444	-0.57	0.042 ^b^	-0.78	0.002 a
Strength of the left arm (Kgf)	-0.10	0.739	-0.048	0.877	-0.03	0.900	-0.50	0.080
Maximum oxygen volume (ml ^−1^ kg ^−1^ min ^−1^ )	0.20	0.509	0.370	0.214	-0.27	0.372	0.06	0.845
**Concerns about the Arm Dimension**								
Body mass index (Kg/m ^2^ )	-0.27	0.369	0.11	0.722	0.50	0.083	0.09	0.766
Weight (kg)	-0.24	0.426	0.10	0.733	0.47	0.106	0.16	0.604
Waist hip ratio (cm/cm)	0.08	0.799	-0.15	0.633	0.68	0.011 a	0.11	0.728
Percentage of fat (%)	-0.43	0.145	0.14	0.648	-0.33	0.264	0.14	0.653
Circumference of the waist (cm)	-0.27	0.378	0.12	0.699	-0.66	0.014 a	-0.37	0.210
Fat-free mass (Kg)	-0.05	0.868	0.07	0.831	0.67	0.011 a	0.09	0.759
Strength of the right arm (Kgf)	-0.33	0.265	0.20	0.510	0.06	0.844	-0.64	0.019 a
Strength of the left arm (Kgf)	-0.24	0.432	0.08	0.801	0.09	0.781	-0.41	0.159
Maximum oxygen volume (ml ^−1^ kg ^−1^ min ^−1^ )	0.20	0.509	0.28	0.361	-0.64	0.018 a	-0.16	0.600
**Body Concerns Dimension**								
Body mass index (Kg/m ^2^ )	-0.10	0.739	0.01	0.964	0.27	0.373	0.01	0.964
Weight (kg)	-0.10	0.741	0.14	0.638	0.37	0.218	0.13	0.678
Waist hip ratio (cm/cm)	0.38	0.198	0.18	0.554	-0.04	0.897	-0.09	0.765
Percentage of fat (%)	-0.37	0.216	0.05	0.868	0.65	0.016 a	0.65	0.016 a
Circumference of the waist (cm)	-0.03	0.927	0.10	0.751	-0.66	0.014 a	-0.37	0.210
Fat-free mass (Kg)	0.14	0.649	0.17	0.586	0.08	0.790	-0.18	0.563
Strength of the right arm (Kgf)	-0.20	0.509	-0.47	0.106	-0.29	0.337	-0.25	0.420
Strength of the left arm (Kgf)	-0.20	0.522	-0.21	0.482	-0.03	0.931	-0.22	0.473
Maximum oxygen volume (ml ^−1^ kg ^−1^ min ^−1^ )	0.20	0.509	0.28	0.361	-0.37	0.220	0.25	0.246
**Transparency Dimension**								
Body mass index (Kg/m ^2^ )	0.10	0.757	0.37	0.211	0.27	0.364	-0.06	0.834
Weight (kg)	-0.08	0.789	0.28	0.360	0.27	0.371	0.04	0.889
Waist hip ratio (cm/cm)	0.31	0.302	-0.24	0.434	-0.10	0.745	0.05	0.866
Percentage of fat (%)	0.07	0.828	0.45	0.126	0.78	0.002 a	0.14	0.647
Circumference of the waist (cm)	0.03	0.920	0.18	0.563	-0.66	0.014 a	-0.37	0.210
Fat-free mass (Kg)	-0.15	0.624	0.10	0.754	-0.08	0.786	-0.03	0.922
Strength of the right arm (Kgf)	0.02	0.953	0.45	0.125	-0.17	0.574	-0.59	0.035 ^b^
Strength of the left arm (Kgf)	0.01	0.996	0.26	0.392	0.08	0.795	-0.35	0.242
Maximum oxygen volume (ml ^−1^ kg ^−1^ min ^−1^ )	0.20	0.509	0.28	0.361	-0.30	0.328	0.05	0.869
**Vulnerability Dimension**								
Body mass index (Kg/m ^2^ )	-0.29	0.341	-0.61	0.027 a	0.22	0.463	0.20	0.519
Weight (kg)	-0.37	0.216	-0.63	0.021 a	0.28	0.362	0.31	0.302
Waist hip ratio (cm/cm)	0.18	0.564	-0.64	0.018 a	0.06	0.851	-0.01	0.963
Percentage of fat (%)	-0.33	0.275	-0.41	0.169	0.57	0.042 ^b^	0.38	0.197
Circumference of the waist (cm)	-0.24	0.428	-0.61	0.026 a	-0.66	0.014 a	-0.37	0.210
Fat-free mass (Kg)	-0.26	0.383	-0.68	0.011 a	0.01	0.981	0.12	0.696
Strength of the right arm (Kgf)	-0.43	0.139	-0.20	0.510	-0.38	0.199	-0.54	0.058
Strength of the left arm (Kgf)	-0.43	0.141	-0.12	0.689	0.16	0.598	-0.24	0.434
Maximum oxygen volume (ml ^−1^ kg ^−1^ min ^−1^ )	-0.46	0.119	0.28	0.361	-0.55	0.053	0.21	0.496

a Strong and significant correlation (r:0.6 the 0.8); ^b^ Moderate and significant correlation (r:0.4 the 0.6); TG: Training Group; CG: Control Group.

The concern with the arm dimension in the CG presented a positive and strong correlation with the waist hip ratio (p = 0.011) and fat-free mass (p = 0.011), and negative correlation with the volume of oxygen (p = 0.018) in the basal period. On the other hand, body composition variables, specifically the BMI (p = 0.027), weight (p = 0.021), waist hip ratio (p = 0.018), WC (p = 0.026), and fat-free mass (p = 0.011) were negatively correlated with V ( Table 3 ).

## Discussion

The main findings of this research were: 1)CT favored positive changes in BI; 2)Variables related to appearance were correlated with the vulnerability dimension in TG; 3)Variables related to body appearance and function were directly related to BI in the CG; 4)CT promoted improvements in functional capacity (FC), but not in body composition in TG women.

## Statement of Main Finding

The central axis of this research is the investigation of BI changes. This was analyzed from the theoretical model with six dimensions 20 and validated for Brazilian women with breast cancer. 3 Three dimensions demonstrated sensitivity to CT: L, BC, and T. After the intervention, the TG demonstrated a reduction in the perception of functional limitations of the body, such as movement restrictions and oncologic fatigue. Arab et al. 13 in one of the few studies in this scope performed in Brazil, presented similar results, although applied only resistance training over 12 weeks. The authors attribute the improvement to the higher physical competence acquired for the performance of motor tasks. Unlike our data, did not find training effects in the other dimensions. 13 It is possible that this difference is associated with the specificity of the intervention given.

Concern with the body is a striking feature in women with breast cancer, 34 is accentuated either through the chemotherapy or mastectomy process. 35 As we hypothesized, the TG showed a tendency to reduce BC, that is, with their general appearance, including concern about the gain or loss of weight. Previous studies have demonstrated similar results after both strength training 16 and aerobic training, 14 in which improvements in the perception of body appearance and lower concern with weight were detected, respectively.

Concern with appearance is related to the alterations promoted by the disease and treatment, which may be less or more visible. 20 Issues relating to concern with how obvious the disease were denominated transparency by the authors. This variable was different between the groups so that women who did not receive the intervention with the CT presented higher scores in this dimension. We did not find similar studies that addressed this point, which limits our discussion. However, a qualitative research with Latina women with breast cancer, identified that the acceptance of changes in appearance is considered a central axis in BI. 9 The authors encourage the development of intervention strategies that favor the acceptance of appearance during and after treatment. Our results suggest that CT may be of potential assistance. This becomes more consistent when we observe the gross scores of all analyzed dimensions and find that, although there is no statistical significance, there is an increase in the CG and reduction in the TG, indicating a tendency to reduce BI impairment with the practice of CT. 3

## Discuss Essential Differences in Comparison to Other Studies

Next question was to identify whether improvements in BI could be attributed to changes in appearance and/and functionality as a result of CT. This hypothesis was partially rejected because the TG did not present a significant correlation between BI and FC. On the other hand, the body composition, BMI, weight, waist hip ratio waist hip ratio, WC, and fat-free mass variables, although not presenting significant changes in our sample, were negatively correlated with BI, specifically with the vulnerability dimension, in the basal period, assuming statistical significance after the intervention. This fact leads us to reflect that the body experience with the CT may have directed the attention of these women to their body measurements, however, differently to women without breast cancer, since the literature indicates that BMI, waist hip ratio, WC, and fat-free mass are predictors of negative changes in BI, such as body dissatisfaction. 34 36 In our study, the opposite occurred, the higher these scores, the lower the feeling of invasion of the body by the disease, which may have consequently caused a lower sensation of vulnerability.

Unlike our results did not identify any variable of body composition and/or function capable of mediating the effect of training on the improvements found in BI. 14 16 On the other hand, the positive effect on FC, identified here and in the studies above, is pointed out by the authors as a factor that influences BI, although indirectly. Speck et al. 16 explain that muscular strength provides benefits to the general quality of life and this, in turn, mediates the intervention in the perception of the body. Pinto et al. 14 concluded that the improvement found in patient's self-assessment of their physical condition is consistent with the increase in VO2max, thus indicating a refinement of the patient about her physical condition.

In contrast to the TG, the BI of the women who were not submitted to the intervention was influenced, over time, as much by the variables related to appearance as by body functionality. WC, a variable commonly associated positively with female body dissatisfaction, 34 precisely because it delineates female body forms, manifested itself in an opposite way for all dimensions of BI. Thus, the smaller this variable, the greater the perception of functional limitations, the concern with the arm and with the body, accentuating the feelings of vulnerability, visibility of the disease (transparency), and BS.

The percentage of fat, also considered a predictor of body dissatisfaction in women, 34 especially for the lean body ideal, 36 maintained this characteristic for the CG, in proportion to the BS, BC, T, and V. This may be due to the gradual and complex process of acceptance of changes in appearance from disease and treatment, requiring women to learn and deal with these changes. 9

Functional capacity also presented an influence on CG BI. The CG showed a negative correlation between arm muscle strength and the limitations and transparency dimensions in all phases. Concerns with the arm were positively related (although not significant at baseline) to muscle strength, assuming statistical significance, but negative, after 12 weeks. The opposite occurred between CA and VO2max, 37 whits the hypothesized that women with breast cancer feel empowered psychologically as they become more physically effective. Although we cannot state that the benefits of CT positively and directly impacted BI in the TG, the authors' idea applies in our results, since we observed that impairments in FC were negatively associated with CG BI.

The TG may have benefited from body experiences in the intervention, thus impacting dimensions which, although not evaluated herein, are indicated in the specific literature as linked to this process: cognitive, affective, and behavioral. 8 Interventions with physical exercise can provide the sensation of regaining control of the body itself, which may translate into a greater sense of self-efficacy in other areas of life. 37 Thus, it is possible to infer that CT promotes subjective experiences that go beyond body appearance and function, although indirectly influencing it.

## Discussion of Secondary Outcomes

However, in the current study, there were no positive changes in body mass, percentage of fat, BMI, or other anthropometric indices in either group. It is worth noting that, despite this, the maintenance of these variables already indicates good maintenance, since the disease and its treatment promote negative changes in body composition. 5 Similar results were found in women with breast cancer submitted to strength training, 15 aerobic 14 and combined protocols. 17 On the other hand had a reduction in fat percentage after intervention with CT. 18 This difference may be attributed to the superiority in the intervention time (24 weeks) and method of analysis (bioimpedance), performed by the researchers. However, the multi-frequency electrical bioimpedance analysis method indicates greater precision when performed in a segmental way, due to the morphological variation in the tissues. 38

On the other hand, there was an interaction between time and group for the right thigh and changes in fat-free mass and WC after 12 weeks. Despite the maintenance of these variables, promoting health through physical training, there was an increase in the WC of both groups, which shows a negative trend and can be attributed to the cancer treatment. There is a large incidence of high WC in patients with breast cancer, which is linked to cardiovascular risk; these authors suggest that this population requires adherence to a nutritional program. 39 40 However, the study by Kim et al. 41 demonstrated a reduction in WC after a 12-week CT intervention, this difference may be due to the stage of the treatment, since all the patients were survivors of breast cancer. Further studies are needed to define the type of intervention effects in the reduction of visceral fat in patients in the treatment of breast cancer.

The effect of CT was confirmed by increased arm muscle strength and improvements in VO2max. These results corroborate studies in the literature which submitted women with breast cancer to protocols composed of aerobic and strength exercises and found similar results. 17 18 19 CT prevents the physical deconditioning inherent in cancer treatment. 19 These variables enhance the perception of this population of improvements in their quality of life. 15 CT promotes greater adherence to physical activity, due to the diversification of exercises. 18

## Strengths and Weakness of the Study

We recognize some methodological limitations of this research. The high exclusion of participants may negatively impact the results of randomized clinical trials, biasing the research. Also, the history of physical exercise of non-eligible patients was not investigated, information that may be useful for understanding some results.

Although we analyzed important variables in the elaboration of the BI of this population, such as the type of surgery, 2 11 a relevant point in this context is breast reconstruction since it is known that women undergoing reconstruction are less dissatisfied with their bodies. 35 Accordingly, we recommend new studies that compare women with and without breast reconstruction and analyze the relationship of BI with body composition and FC.

## Clinical Implications

Thus, the data obtained here have theoretical and practical implications refers to the need to broaden the understanding of BI adaptations as a function of specific physical changes in breast cancer, using a specific tool and theoretical axis that considers BI as a multidimensional and independent construct.

We believe that this information could help in the delineation of facilitating factors, mediators, and protectors of BI in the treatment process, allowing the elaboration of adequate interventions. Practical implications involve the use of this information in interventions that deal directly with the body, making them more assertive and efficient.

## Conclusion

CT was shown to be a useful strategy capable of promoting improvements in the FC and BI of women with breast cancer. The effect of combined training may imply improvements in these variables that are negatively related in cancer patients who do not undergo physical training. Thus, we recommend the combined training use together with conventional treatment.
